# Politikberatung durch den Deutschen Ethikrat: Ein Kommentar zur argumentativen Qualität von normativen Empfehlungen zur Bewältigung der Covid-19 Pandemie

**DOI:** 10.1007/s41358-021-00282-3

**Published:** 2021-08-25

**Authors:** Sven Grundmann

**Affiliations:** Friedrich-Alexander Universität Erlangen, Room 02.274, Henkestraße 91, Haus 8, 91052 Erlangen, Deutschland

Die Funktion des Deutschen Ethikrats und seinem Vorläufer, dem Nationalen Ethikrat, ist die Politikberatung im Zusammenhang mit normativen Konflikten auf dem Gebiet der Lebenswissenschaften. Im Ethikratgesetz heißt es hierzu:

Der Deutsche Ethikrat verfolgt die ethischen, gesellschaftlichen, naturwissenschaftlichen, medizinischen und rechtlichen Fragen sowie die voraussichtlichen Folgen für Individuum und Gesellschaft, die sich im Zusammenhang mit der Forschung und den Entwicklungen insbesondere auf dem Gebiet der Lebenswissenschaften und ihrer Anwendung auf den Menschen ergeben. (§ 2, Abs. 1 EthRG)

Gordian Ezazi (2015) weist darauf hin, dass es im Gesetzestext keine Hinweise gibt, ob die Rangfolge der angeführten Aufgaben einer Hierarchisierung von Prioritäten entspricht. Auch der Gegenstand der geforderten Beratungstätigkeit ist nicht genau definiert.

Zur Stellung des Deutschen Ethikrats im politischen Prozess gibt es unterschiedliche Deutungen. Kathrin Braun (2013) untersucht den Deutschen Ethikrat aus einer machttheoretischen Perspektive im Sinne von Foucault. Politische Ethikräte erscheinen ihr als eine politische Technologie, durch die unentscheidbare politische Fragen politisch entscheidbar gemacht werden sollen. Die inhaltlichen Stellungnahmen zu moralischen Konflikten sind für den politischen Prozess aus ihrer Sicht jedoch weniger relevant. Wichtig erscheint Braun vor allem, dass der Ethikrat stattfindet und durch seine spezifische Art des Abwägens, Erwägens, Deliberierens und Evaluierens vorführt, wie im politischen Diskurs zu sprechen ist.

In eine ähnliche Richtung geht auch die soziologische Perspektive von Barth et al. (2017, S. 291f). In Anlehnung an Luhmann vermuten sie, dass im Rahmen des Ethikrats eine Form der Rede eingeübt wird, die mit multiplen Systemreferenzen zu rechnen lernt. Die Universalitätsansprüche unterschiedlicher Perspektiven werden im Ethikrat als unterschiedliche Universalismen repräsentiert – ohne sie unter den Zwang eines einzelnen guten Grundes und damit einer Entscheidung für eine bestimmte Perspektive zu zwingen.

Alexander Bogner (2011, 2014) untersucht Ethikräte aus einer mikropolitischen Perspektive. Entgegen dem Anliegen der Theorie Kommunikativen Handeln von Habermas sei das Ziel der Beratungsverfahren nicht, zu einem moraltheoretisch begründeten Konsens zu kommen. Das Ergebnis der Beratungen sei, so Bogner, vielmehr ein begründeter Expertendissens. Der Dissens ist jedoch kein Makel, sondern die geordneten Stellungnahmen bieten eine Orientierung für politische Gewissensentscheidungen. Mithin tragen Ethikräte durch ihre Stellungnahmen zu einem politischen Framing bei. Werden Konflikte als Wertkonflikte gerahmt, so werden sie nach Bogner im politischen Diskurs anders verhandelt als etwa Verteilungskonflikte oder Wissenskonflikte.

Während die zuvor genannten Autoren vor allem die „Througput-Legitimation“ des Ethikrats im politischen Prozess betonen, geht es mir im Rahmen dieser Analyse um die konkrete argumentative Qualität der Erzeugnisse des Deutschen Ethikrats im Zusammenhang mit den normativen Stellungnahmen zur Covid-19 Pandemie. Aus einer argumentationstheoretischen Perspektive ist anzunehmen, dass Mehrheitsentscheidungen, die auf der Grundlage von durchdachten und begründeten Erwägungen getroffen werden, welche aus einer Vielzahl von Perspektiven intersubjektiv nachvollziehbar sind, eine höhere Legitimation aufweisen, als solche, die nur ein Ausdruck der Akkumulation unreflektierter Meinungen sind (vgl. u. a. Rawls 1998). Auch wenn es für politische Entscheidungen keine übergeordnete normative Basis gibt, von der aus sich allgemein verbindlich – und für alle Menschen einsichtig – Konflikte rational auflösen lassen, besteht dennoch die Möglichkeit, die Qualität von Argumenten in normativen Stellungnahmen formal zu beurteilen. Damit die Stellungnahmen des Deutschen Ethikrats eine normative Orientierungsfunktion erfüllen, müssen sie höheren formalen Ansprüchen genügen als Sprechakte im Rahmen von bloßen Meinungsäußerungen. Das Modell von Stephen Toulmin (2003) ermöglicht die Qualität von Argumenten auf der Grundlage ihrer strukturellen Eigenschaften zu beurteilen. Wie verschiedene Autoren der Politischen Ethik und Politikberatung zeigen (u. a. Fischer 2006; Fishkin 1991; Bromell 2017), hängt die Qualität normativer Entscheidungen maßgeblich von der Art und Weise ihrer Begründungen ab.

Der Deutsche Ethikrat hat sich seit Ausbruch der Covid-19 Pandemie (im Zeitraum von Dezember 2019 bis Juli 2021) mit einer Stellungnahme und drei Ad-Hoc Empfehlungen zu Wort gemeldet. Die umfangreichere Stellungnahme zu Immunitätsbescheinigungen erschien im September 2020. Die erste Ad-Hoc Empfehlungen, in Zusammenarbeit mit der Leopoldina und der STIKO, wurde im November 2020 veröffentlicht und thematisiert die Regelung des Zugangs zu COVID-19-Impfstoffen. Die zwei weiteren Ad-Hoc Empfehlungen behandeln normative Fragen im Zusammenhang mit sozialen Kontakten in der Langzeitpflege (Veröffentlichung im Dezember 2020) und besonderen Regeln für Geimpfte (Veröffentlichung im Februar 2021).

Im Folgenden werde ich das Argumentationsmodell von Stephen Toulmin (2003) vorstellen und exemplarisch erläutern. Anschließend überprüfe ich die formale Qualität der normativen Stellungnahmen des Deutschen Ethikrats am Beispiel von zwei Publikationen. Die erste Untersuchung betrifft die Stellungnahme zu Immunitätsbescheinigungen. Die zweite hier untersuchte Publikation ist eine Ad-Hoc Stellungnahme und befasst sich mit der zentralen Frage nach einer gerechten Impfreihenfolge. Letztere Publikation entstand in Zusammenarbeit mit Mitgliedern der Ständigen Impfkommission und der Nationalen Akademie der Wissenschaften, Leopoldina. Ich habe mich bewusst für diese beiden Publikation entschieden, weil hier zentrale Fragen des Pandemie-Managements verhandelt werden. Die Stellungnahme zu einer allgemeinen Impfpflicht habe ich hingegen nicht berücksichtigt, da diese Publikation noch vor dem Ausbruch der Covid-19 Pandemie erarbeitet wurde. Nach der Evaluation der normativen Stellungnahmen gemäß Toulmins (2003) Modell für formal robuste Argumente, werde ich die Bedeutung der Ergebnisse für die Stellung ethischer Politikberatung im politischen System reflektieren.

## Argumentationsmodell von Stephen Toulmin

Klar und präzise normative Positionen darzulegen, zu erklären und auf Einwände konstruktiv zu reagieren, hat mit politischen Verlautbarungen wie in TV-Talkshows nur wenig gemein. Damit ein Expertendiskurs, der nicht selten in einem Expertendissens mündet, eine Orientierungsfunktion im politischen und gesellschaftlichen Diskurs bieten kann, muss sichergestellt sein, dass die argumentative Qualität von Argumenten über den medialen „Politikersprech“ hinausgeht. In der medialen öffentlichen Arena haben die Sprecher häufig gar keine Verständigungsabsicht, denn ihnen geht es vor allem darum, aus Stimmungen Stimmen zu generieren (Neidhardt 1994). Robustes Argumentieren ist demgegenüber eine Praxis, die das Ziel der intersubjektiven Verständigung anstrebt. Eine Verständigung wird auch dann angestrebt, wenn keine normative Einigung erzielt werden kann. Strukturell starke Argumente befördern intersubjektive Verständigung, lassen Rückfragen und Erläuterungen zu, statt mittels Manipulation andere Perspektiven zu verdrängen oder umzupolen. Stephen Toulmin (2003) präsentiert in seinem Argumentationsmodell eine Struktur, wie robuste Argumente formal aufgebaut sind. Als Zutaten nennt er sechs Elemente. Diese Elemente sind: claims (1), grounds (2), warrants (3), backing (4), modal qualifications (5) und possible rebuttals (6). Im Folgenden erläutere ich, wie diese sechs Elemente zusammenwirken (siehe hierzu auch Toulmin et al. 1979, S. 25ff):

1. Claims sind Aussagen, beziehungsweise Schlussfolgerungen, deren Richtigkeit wir im Verlauf der Argumentation (im Verbund mit den anderen Elementen) zeigen wollen.
2. Grounds, beinhalten Tatsachenbehauptungen, auf denen Argumente aufbauen. Abhängig vom konkreten Anwendungsbereich kann es sich bei diesen Informationen unter anderem um Allgemeinwissen, eigene Erfahrungen, statistische Daten oder experimentelle Beobachtungen handeln.
3. Warrants sind die Schlussregeln, die eine Verknüpfung zwischen den grounds und den claims herstellen. In den Naturwissenschaften werden warrants als Naturgesetz formuliert, während in der Moraltheorie warrants als verbindliche Normen, zum Beispiel in Gestalt des kategorischen Imperativs, formuliert werden können.
4. Backing bezeichnet eine argumentative Stütze, durch die die Validität der warrants abgesichert wird. In den Naturwissenschaften erfolgt die Autorisierung durch die in experimentellen Testverfahren erhobenen Daten. In der Ethik kann die Autorisierung von Schlussregeln zum Beispiel im Rahmen von Gedankenexperimenten erbracht werden.
5. Modal qualifiers, auch Operatoren genannt, beinhalten Angaben, unter welchen Bedingungen die warrants (Schlussregeln) Geltung beanspruchen und/oder wie stark der angegebene Kausalitätszusammenhang ist. Während die Eintrittswahrscheinlichkeit bei einigen Schlussfolgerungen 100 % beträgt, können für andere Kausalitätszusammenhänge nur probabilistische Aussagen getroffen werden.
6. Possible rebuttals sind Ausnahmebedingungen, unter denen der Kausalitätszusammenhang zwischen grounds und claims nicht zutrifft. Für eine robuste Argumentation müssen kritische Einwände antizipiert und in der eigenen Argumentation reflektiert werden.

**Figure 1:**
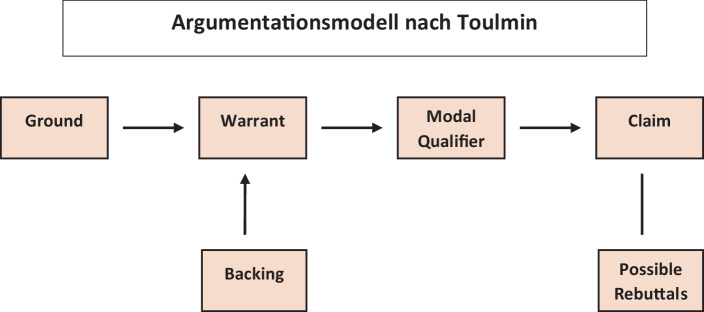

Abb. 1 gibt einen Überblick zum Zusammenwirken der besprochenen Elemente im Rahmen von Toulmins Argumentationsmodell für formal robuste Argumente.

Formal robuste Argumente werden in der Regel mit einer Tatsachenbehauptung (ground) eingeleitet, welche die für die eigene Schlussfolgerung notwenigen Daten enthält. Die Tatsachenbehauptung ist mit einer Schlussregel (warrant) verknüpft. Beide Elemente stellen einen Zusammenhang her und münden in einer Schlussfolgerung (claim). Die Schlussregel (warrant) wird durch zusätzliche Informationen (backing) abgestützt, welche die Gültigkeit der Schlussregel autorisieren. Zusätzlich zu diesen Kernelementen können modal qualifiers angeben, unter welchen Bedingungen, und wie zuverlässig die Schlussregel Geltung beansprucht. Darüber hinaus werden mögliche Gegenargumente (possible rebuttals) reflektiert und mögliche Umstände genannt, unter denen die Argumentationskette zusammenbricht (Grundmann 2020). Am Beispiel eines Arguments zur Einführung einer Steuer auf gesundheitsschädliche Lebensmittel aus den USA, werde ich den Aufbau formal robuster Argumente exemplarisch erläutern (Tab. 1).

**Table 1:** 

Ground	Die Amerikaner nehmen heute fast 20 % mehr Kalorien zu sich als in den frühen 1980er-Jahren. Ein Großteil dieser Kalorien stammt von stark verarbeiteten fett- und zuckerreichen Lebensmitteln. Hinzu kommt, dass verarbeitete Lebensmittel und Getränke häufig erschwinglicher sind als Obst, Gemüse und Vollkornprodukte, die für eine gesunde Ernährung unabdingbar sind
Claim	Eine Steuer auf ungesunde Lebensmittel kann dazu beitragen, das Problem der Fettleibigkeit zu bekämpfen
Warrant	Die Kaufentscheidungen für Lebensmittel hängen maßgeblich von deren Kosten ab. Aus diesem Grund kann eine Steuer auf ungesunde Lebensmittel eine steuernde Wirkung entfalten
Backing	Wie Steuern auf gesundheitsschädliche Produkte die öffentliche Gesundheit positiv beeinflussen können, demonstriert das Beispiel der Einführung einer Tabaksteuer in New York. Die Raucherquote sank dort nach der Einführung dieser Steuer um 12 Prozentpunkte auf einen historischen Tiefpunkt. Die positiven Wirkungen von Tabaksteuern lassen auf eine positive Wirkung einer „Junk-Food-Steuer“ schließen
Rebuttal	Ein Steuer auf ungesunde Lebensmittel kann Menschen aus einkommensschwachen Haushalten zusätzlich belasten, da gesunde Lebensmittel in der Regel teurer sind und in armen Gegenden vor Ort häufig weniger verfügbar. Zudem könnte gegen das Argument eingewandt werden, dass der Konsum von Tabak eher der Wahl eines Lebensstils entspricht, als es dies bei der Auswahl von Lebensmitteln der Fall ist
Qualifier	Gesunde Lebensmittel müssen für die Bevölkerung leistbar und vor Ort verfügbar sein, da nur so eine positive Veränderung des Konsumverhaltens erwartbar ist. Aus diesem Grund muss gewährleistet werden, dass eine gesunde Ernährung in allen Bezirken möglich ist. Mit dem „Zuckerbrot“ subventionierter gesunder Lebensmittel und der „Peitsche“ einer Lebensmittelsteuer kann die Verbesserung der Öffentlichen Gesundheit dann vorangetrieben werden

^a^Das Beispiel habe ich einer Übung des Schreibzentrums der Universität Richmond entnommen (http://writing2.richmond.edu/writing/wweb/toulminexercise.html)

## Die Stellungnahmen des Deutschen Ethikrats zu Immunitätsbescheinigungen in der Covid-19 Pandemie

Die Stellungnahme zu Immunitätsbescheinigungen in der Covid-19 Pandemie wurde im September 2020, als es noch keine am Markt zugelassenen Impfstoffe gab, veröffentlicht (Deutscher Ethikrat 2020a). Die im Vergleich zu den Ad-Hoc Empfehlungen umfangreichere Stellungnahme folgt weitgehend der wiederkehrenden Struktur für Stellungnahmen des Deutschen Ethikrats. Stellungnahmen des Deutschen Ethikrats enthalten in der Regel folgende Komponenten (vgl. Ezazi 2015; Grundmann 2020):

- Der naturwissenschaftlich-medizinische Teil beinhaltet eine Zusammenfassung des aktuellen wissenschaftlichen Sachstands. Die bereitgestellten Informationen enthalten Definitionen von medizinischen Grundbergriffen, Erklärungen zu verschiedenen Forschungsansätzen und Erläuterungen zu den zu diskutierenden medizinischen Verfahren.
- Der rechtliche Teil definiert die relevanten Rechtsbegriffe und verweist auf das geltende Recht. Darüber hinaus wird der Gesetzgeber auf mögliche rechtspolitische Implikationen hingewiesen.
- Der ethische Teil arbeitet die normativen Konfliktlinien heraus. Welche Werte gilt es in der Reflexion zu berücksichtigen, beziehungsweise zwischen welchen Werten muss eine Entscheidung getroffen werden? Am Ende dieses Teils werden politische Handlungsempfehlungen gegeben. Diese können je nach Ergebnis der Beratungen unterschiedlich ausfallen: eine einstimmige Empfehlung im Konsens oder abweichende Handlungsempfehlungen durch divergierende Voten in Form von Mehrheits- und Minderheitsvotum. Die Ratsmitglieder haben zudem die Option ein Sondervotum abzugeben.

Abweichend von der oben genannten Struktur enthält die Stellungnahme zu Immunitätsbescheinigungen in der Covid-19 Pandemie keinen expliziten rechtlichen Teil. Nach dem naturwissenschaftlich-medizinischen Teil folgen Abschnitte zu den normativen Positionen und ihren Empfehlungen. In der Stellungnahme geht es um die Frage, ob es staatliche Immunitätsbescheinigung geben sollte. Das heißt, sollen jene Menschen, welche die Covid-19 Infektion überstanden und in ihrem Körper Antikörper gebildet haben, von den Eingriffen in ihre Freiheitsrechte ausgenommen werden (Entzug der Freiheitsrechte durch Einschränkung der Versammlungsfreiheit, Verpflichtung zum Tragen eines Mund-Nasenschutzes). Die Position A empfiehlt Maßnahmen, die nach erfolgter Genesung im Rahmen von Immunitätsbescheinigungen die Rechtsbeeinträchtigungen stufenweise aufheben und eventuell mit bestimmten Pflichten verbinden. Position B kommt demgegenüber zu einer negativen Einschätzung und hält Immunitätsbescheinigungen (und damit verbundene Aufhebungen von Beschränkungen) selbst dann für unverantwortbar, wenn eine Immunität und Nichtinfektiosität von Genesenen zweifelsfrei und zuverlässig nachweisbar ist. Im Folgenden überprüfe ich, ob die normativen Positionen A und B gemäß des Argumentationsmodells von Toulmin als robuste Argumente gelten können.

Wie Tab. 2 zeigt, entspricht die Argumentationskette der normativen Position A vollumfänglich den formalen Anforderungen robuster Argumentation nach Toulmin. Die Schlussregel, das heißt die Aussage, dass der Legitimationsgrund für Freiheitsbeschränkungen entfällt, sofern von Personen nachweislich kein Infektionsrisiko ausgeht, wird mit zusätzlichen Argumenten gestützt. Mit der Freiheitsrückgewähr, so die Argumentation, sind Schadensbegrenzungen im Sinne des Gemeinwohls verbunden. Für eine noch solidere Abstützung des Arguments hätte auf konkrete Gesetztestexte, oder auf mögliche Urteile des Bundesverfassungsgerichts verwiesen werden können, die eindeutig belegen, dass nicht individuelle Freiheitsbetätigungen, sondern hoheitliche Freiheitsbeeinträchtigungen rechtfertigungsbedürftig sind. Positiv ist zu erwähnen, dass mögliche Einwände gegen Immunitätsbescheinigungen (soziale Spaltungen und Stigmatisierungen zwischen immunen und nicht-immunen Personen) in der Argumentation adressiert werden. Die Kritikpunkte seitens der Gegner von Immunitätsbescheinigungen werden im weiteren Verlauf in Zusammenhang mit möglichen Strategien zu deren Bewältigung diskutiert. Ein Vorschlag lautet, dass mit der Wiedererlangung von Rechten auch Pflichten für immune Personen einhergehen können. Verweise auf weiterführende Literatur verleihen den Aussagen zusätzliches Gewicht. Allerdings besteht in diesem Punkt noch Verbesserungspotential. Gibt es eventuell Studien, die empirisch belegen, dass es durch den staatlich verordneten Lockdown zu einer Zunahme von häuslicher Gewalt kommt? Mit Verweis auf wissenschaftliche Studien oder amtliche Zahlen würden solche Aussagen ihren spekulativen Charakter verlieren.

**Table 2:** 

Ground	Die Darstellung der Faktenlage hinsichtlich des wissenschaftlichen Sachstands ist identisch mit Position B. Allerdings werden verschiedene Aspekte unterschiedlich betont. „Während Tests, die eine akute Infektion nachweisen (PCR- oder Antigentests), nur eine Momentaufnahme der akut vorhandenen Viruskonzentration darstellen, spiegeln Antikörpertests eine längerfristige Antwort des Immunsystems auf einen Erreger wider. Auch zurückliegende Infektionen können so erkannt werden. Die Tests erfassen Antikörper, die eine erkrankte Person im Laufe der auf die Infektion folgenden Wochen und Monate entwickelt und die über Jahre im Blut nachweisbar sein können. Sollte künftig bekannt werden, dass eine gewisse Konzentration bestimmter Antikörper ausreichend Schutz vor einer erneuten Erkrankung an Covid-19 und der Ansteckung anderer Menschen verleiht, könnte sich ein entsprechender Antikörpernachweis als Grundlage für eine etwaige Immunitätsbescheinigung eignen“ (Deutscher Ethikrat 2020a, S. 14)
Claim	Infektionsschutzbedingte Grundrechtsbeschränkungen können im Rahmen einer Immunitätsbescheinigung zurückgenommen werden, sofern sichergestellt ist, dass von der immunisierten Bevölkerung keine Selbst- und Fremdgefährdung ausgeht. Zudem bieten Immunitätsbescheinigungen die Chance, das Gemeinwohl zu steigern (Deutscher Ethikrat 2020a, S. 20)
Warrant	Maßnahmen zur Wiedererlangung individueller Freiheiten sind stattzugeben, sofern der Gesundheitsschutz und die Pandemiebekämpfung gewährleistet sind und mit einer Steigerung des Gemeinwohls gerechnet werden kann. „Ein solches Vorgehen entspräche der allgemeinen rechtsstaatlichen Maßgabe, dass nicht individuelle Freiheitsbetätigungen, sondern hoheitliche Freiheitsbeeinträchtigungen rechtfertigungsbedürftig sind. Wo der bisherige Legitimationsgrund entfallen ist – etwa, weil von einer Person nachweislich kein Infektionsrisiko mehr ausgeht –, darf eine Beschränkung deshalb grundsätzlich nicht aufrechterhalten werden. Eine Rücknahme von Freiheitseinschränkungen ist daher nicht per se als diskriminierend anzusehen. Der Nachweis einer Immunität und die daraus resultierende relative Nichtgefährdung für sich und andere würde vorbehaltlich anderer für die Aufrechterhaltung der Maßnahme sprechender Gründe prinzipiell eine Ungleichbehandlung rechtfertigen“ (Deutscher Ethikrat 2020a, S. 24)
Backing	„Mit dieser Freiheitsrückgewähr wären zugleich Schadensbegrenzungen verbunden. Die infektionsschutzbedingten Maßnahmen führen seit ihrer Einführung zu erheblichen Beeinträchtigungen für nahezu alle Mitglieder der Gesellschaft, in vielen Fällen und bei zunehmender Dauer sogar mit schwerwiegenden Begleitschäden. Hierzu zählen nicht nur erhebliche Einschränkungen elementarer persönlicher wie politischer Freiheitsrechte. Negative Folgen entstehen auch in bildungsbezogener, psycho-sozialer, kultureller und wirtschaftlicher Hinsicht. Selbst unmittelbar gesundheitsrelevante Begleitschäden sind in diesem Zusammenhang zu beachten, beispielsweise aufgeschobene Operationen, nicht wahrgenommene ärztliche Versorgung, medizinisch-therapeutische Unterversorgung von Personen, die etwa in Einrichtungen der Alten- und Behindertenhilfe mit stärkeren Zugangsbeschränkungen leben, Isolation und Vereinsamung allein lebender Personen im häuslichen Umfeld, stressinduzierte häusliche Gewalt usw. Die gelegentlich bemühte Frontstellung zwischen Gesundheitsschutz auf der einen und Wirtschaftsschutz auf der anderen Seite geht fehl, weil wirtschaftliche, gesundheitliche und andere soziale Güter nicht getrennt voneinander betrachtet werden können“ (Deutscher Ethikrat 2020a, S. 24f)
Rebuttal	Für einzelne Personen könnte die (Wieder‑)Erlangung von Freiheit im Rahmen von Immunitätsbescheinigungen ein Anreiz sein, sich selbst zu infizieren, um die Vorteile von immunisierten Personen zu erlangen. Durch Immunitätsbescheinigung könnte zudem die Bereitschaft zur Regelbeachtung von Schutzmaßnahmen innerhalb der restlichen Bevölkerung sinken, da Immunität von außen nicht erkennbar ist. Die Einführung von staatlichen Immunitätsbescheinigungen könnte gegenüber nicht immunisierten Personen, etwa in prekären Beschäftigungsverhältnissen, auch zu neuen Formen der Exklusion und Stigmatisierungen führen und soziale Spannungen verstärken (Deutscher Ethikrat 2020a, S. 21ff.)
Qualifier	„Den erwähnten Risiken, wie etwa sozialer Ausgrenzung und einem Anreiz zur Selbstinfektion, ist durch eine sorgfältige und kontextbezogene Prüfung der Auswahl rückgewährter Freiheitsrechte vorzubeugen. Es kann gegebenenfalls auch in Betracht gezogen werden, mit der Ausstellung einer Immunitätsbescheinigung bestimmte Pflichten zu verbinden“ (Deutscher Ethikrat 2020a, S. 24). „Um der Gefahr der Selbstinfektion zu begegnen, müsste die Einführung von Immunitätsbescheinigungen zudem mit einer bundesweiten Aufklärungsinitiative zu den Gefahren von Covid-19 verbunden werden“ (Deutscher Ethikrat 2020a, S. 26)

In Tab. 3 führe ich die formale Argumentationsanalyse für die normative Position B durch.

**Table 3:** 

Ground	Die Darstellung der Faktenlage hinsichtlich des wissenschaftlichen Sachstands ist identisch mit Position A, allerdings werden verschiedene Aspekte in den normativen Positionen unterschiedlich betont: „Aktuell befindet sich eine Vielzahl frei zugänglicher Antikörpertests auf dem Markt, die sich in ihrer Funktionsweise und Qualität sehr stark unterscheiden und oftmals eine sehr hohe Unsicherheit aufweisen. Tests, die keinen zuverlässigen Nachweis neutralisierender Antikörper liefern oder aufgrund mangelnder Spezifität viele falsch-positive Ergebnisse erzielen, könnten Immunität suggerieren, die in Wahrheit nicht vorhanden ist. Verhalten sich Personen mit einem falsch-positiven oder anderweitig wenig aussagekräftigen Testergebnis nun so, als ob sie immun wären, (…) setzen sie sich (und potenziell auch Menschen in ihrem Umfeld) einem erhöhten Infektionsrisiko aus“ (Deutscher Ethikrat 2020a, S. 16f, vgl. S. 41)
Claim	Selbst „für den Fall, dass es künftig gesicherte Erkenntnisse über eine länger anhaltende Immunität sowie hinreichend zuverlässige Tests zum Nachweis von Immunität und Nichtinfektiosität geben sollte, sprechen gewichtige praktische, ethische und rechtliche Gründe gegen die Einführung von staatlich kontrollierten Immunitätsbescheinigungen“ (Deutscher Ethikrat 2020a, S. 38)
Warrant	„Es ist also nicht nur die unterschiedliche Bewertung des wissenschaftlichen Sachstandes, sondern vor allem auch eine andere normative Grundierung, die dazu führt, dass Position A und B zu einer unterschiedlichen Bewertung des Instruments Immunitätsbescheinigung und folglich auch zu anderen Empfehlungen kommen“ (Deutscher Ethikrat 2020a, S. 38)
Backing	„Mit Blick auf Gerechtigkeitsfragen sind bei einer Koppelung von Rechten oder Pflichten an den Status der Immunität ungerechte Verteilungen von Chancen, aber auch von Risiken, Belastungen und Einschränkungen in zwei Richtungen möglich: Einerseits, wenn Personen ohne Immunitätsbescheinigung Möglichkeiten verwehrt würden (zum Beispiel der Besuch einer Ausbildungsstätte); andererseits, wenn Personen mit Immunitätsbescheinigung für bestimmte Tätigkeiten besonders in die Pflicht genommen würden (zum Beispiel medizinisches Personal, Reinigungskräfte, Verkaufspersonal, Personal in Kindertagesstätten oder Schulen)“ (Deutscher Ethikrat 2020a, S. 40). „Die (…) mit der Einführung einer freiheitsgewährleistenden Immunitätsbescheinigung verbundenen Probleme stellen eine große Herausforderung für einen angemessenen Rechtsrahmen dar, die kaum praktisch-politisch zu bewältigen ist (…). Das liegt zum einen an der Dynamik demokratischer Entscheidungsprozesse, zum anderen aber auch an Partikularinteressen derer, die von Immunitätsbescheinigungen profitieren würden. Zugleich sind diejenigen, die von Gefahren und Nachteilen des Instruments betroffen wären, politisch weniger stark repräsentiert. Dies betrifft beispielsweise notwendige gesetzliche Anpassungen in Bezug auf Missbrauchsgefahren im privatwirtschaftlichen Kontext, hinsichtlich des Datenschutzes sowie des Arbeitsrechts“ (Deutscher Ethikrat 2020a, S. 44f)
Rebuttal	Im Rahmen der Position A werden zahlreiche Gegenargumente, das heißt Argumente, die für Immunitätsbescheinigungen sprechen, dargelegt. In der Begründung der normativen Position B werden diese teilweise aufgegriffen: „Auch der Wunsch nach (Dienst‑)Planungssicherheit durch Immunitätsbescheinigungen in Gesundheits- und Pflegebereichen oder in Schulen und Kindertagesstätten ist zwar dem Grunde nach nachvollziehbar. Allerdings ist davon auszugehen, dass in den Einrichtungen erheblicher Druck gegenüber dem Personal erzeugt werden würde, sich einem Antikörpertest zu unterziehen, um eine Immunitätsbescheinigung zu erhalten und damit Aufgaben mit einem höheren Risikopotenzial übernehmen zu können“ (Deutscher Ethikrat 2020a, S. 41). (…) „Zweifelsohne wäre die Kenntnis über eine Immunität und Nichtinfektiosität für jede Person eine große Entlastung. Das gilt insbesondere für diejenigen, die in ihrem Beruf oder in ihrem Alltag zwangsläufig einem besonderen Infektionsrisiko ausgesetzt sind. Wenn aber eine Immunitätsbescheinigung keine ausreichend gesicherte Grundlage aufweist, würde sie diese Personen in einer gefährlichen Scheinsicherheit wiegen und erhebliche Risiken für sie selbst und für andere bergen“ (Deutscher Ethikrat 2020a, S. 41)
Qualifier	„Sollten sich Antikörpertests in Zukunft wider Erwarten als hinreichend zuverlässig erweisen, so sollten diese nur in streng definierten Einzelfällen zur individuellen Rückgewähr von Freiheit oder zur Auferlegung besonderer Verpflichtungen genutzt werden dürfen. Lediglich zugunsten besonders vulnerabler Gruppen, die etwa in Einrichtungen der Alten- oder Behindertenhilfe erheblich unter den strengen Isolationsmaßnahmen zu leiden haben, dürften nahe An- und Zugehörige, gegebenenfalls auch ehren- oder hauptamtliche Angehörige begleitender externer Dienste (Seelsorger, Hospizdienste usw.) auf der Grundlage gesicherter Kenntnis über ihre Immunität und Nichtinfektiosität von bestimmten Auflagen befreit werden. Solche Maßnahmen machen allerdings keine staatliche Immunitätsbescheinigung erforderlich, sondern ließen sich im Infektionsschutzgesetz etwa durch eine Vorschrift verbindlich regeln, wonach Ärzte ermächtigt werden, für diese Personengruppe auf der Basis entweder eines hinreichend und zuverlässigen aktuellen PCR-Tests oder aber eines – gegebenenfalls in Zukunft zur Verfügung stehenden – hinreichend zuverlässigen Antikörpertests, eine entsprechende Bescheinigung der – hochwahrscheinlichen – Nichtinfektiosität auszustellen“ (Deutscher Ethikrat 2020a, S. 42)

Auch die Positionen B, die sich gegen Immunitätsbescheinigungen ausspricht, entspricht in ihrem formalen Aufbau weitgehend einem robusten Argument nach Toulmin. Wie die Autoren der Position B betonen, ist für die Begründung ihrer Bewertung nicht eine unterschiedliche Bewertung des Sachstands maßgeblich, sondern eine von der Position A abweichende normative Grundierung. Allerdings ist die normative Argumentationskette, die in einer negativen Bewertung von Immunitätsbescheinigungen mündet, nur in Umrissen zu erkennen. Als Schlussregeln werden ethische und rechtliche Probleme benannt. Als ein ethisches Problem erscheint in dieser Perspektive die Ungleichbehandlung zwischen immunen und nicht-immunen Menschen. Es bleibt jedoch offen, welcher Gerechtigkeitsgrundsatz durch Immunitätsbescheinigungen genau verletzt wird und worauf sich die normative Geltung dieses Grundsatzes stützt. Auch die angeführten rechtlichen Bedenken bleiben sehr vage und lassen im Rahmen einer „Textbefragung“ keine weiteren Rückfragen zu. Als Problem wird beispielsweise die Dynamik politischer Entscheidungsprozesse und Partikularinteressen angeführt, welche sich, so die Autoren der Position B, im Gesetzgebungsprozess kaum bewältigen ließen. Was damit genau gemeint ist und worauf sich diese Behauptung stützt, bleibt unklar. Zudem wird, ohne einen argumentativen Zusammenhang herzustellen, auf arbeitsrechtliche Bedenken und Probleme mit dem Datenschutz verwiesen. Auch hier wird nicht genau begründet, warum Immunitätsbescheinigungen aus einer datenethischen Perspektive problematisch sind. Die formalen Schwächen in der Argumentation treten zudem durch die unzureichende Berücksichtigung von Gegenargumenten in Erscheinung. So werden die Einwände der Position A in der Reflexion möglicher Ausnahmebedingungen (rebuttals) kaum berücksichtigt. Die für Position A zentrale Schlussregel (warrant), dass nicht individuelle Freiheitsbetätigungen, sondern hoheitliche Freiheitsbeeinträchtigungen rechtfertigungsbedürftig sind, wird von den Vertretern der Position B gänzlich ignoriert. Auf welches Recht und welche normative Grundlage sich demgegenüber die Position B beruft, geht aus der Stellungnahme nicht hervor.

Über die formalen Defizite hinaus, könnte die Argumentation der Position B verbessert werden, indem wichtige Aussagen mit Verweisen auf wissenschaftliche Quellen und mit fundierten Daten belegt werden würden. So behaupten die Vertreter der Position B, dass das Deutsche Gesundheitssystem mit der Ausstellung von Immunitätsbescheinigungen überfordert werden könnte, so dass für erfolgversprechendere Maßnahmen des Pandemie-Managements, wie beispielsweise eine verbesserte Versorgung mit Schutzkleidung und -masken in Pflegeheimen, keine Mittel mehr bereitstünden (Deutscher Ethikrat 2020a, S. 44). Dass der größten Volkswirtschaft Europas das Geld für Schutzbekleidung ausgeht, wenn im Zuge der Einführung von Immunitätsbescheinigungen mehr Antikörpertests durchgeführt werden müssen, erscheint in Anbetracht der Konjunkturprogramme in Milliardenhöhe als eher fragwürdig.

## Gemeinsame Ad-Hoc Empfehlung zum Zugang zu Covid-19 Impfstoffen

Neben den umfassenderen Stellungnahmen veröffentlicht der Deutsche Ethikrat auch Ad-Hoc Empfehlungen zu aktuellen politischen Debatten. Im Gegensatz zu den längeren Stellungnahmen haben Ad-Hoc Empfehlungen einen begrenzten Umfang von nur wenigen DIN A4-Seiten. Der inhaltliche Aufbau dieser Ad-hoc-Empfehlungen entspricht, wie Ezazi (2015, S. 87) feststellt, „erkennbar jenem einer standardüblich publizierten Stellungnahme: Einer thematischen Hinführung folgt die Wiedergabe des naturwissenschaftlichen Sachstandes, die Erläuterung der rechtlichen Prämissen und zum Schluss eine einstimmig von den Mitgliedern des Rates geteilte politischen Empfehlung“. Allerdings gibt es in den Ad-hoc-Empfehlungen keinen ethischen Reflexionsteil, in dem normative Forderungen ausführlich begründet werden. Wahrscheinlich wird dieser Teil als verzichtbar angesehen, da konsensual verabschiedete Empfehlungen ausgesprochen werden. Abweichende Minderheits- oder Sondervoten werden allenfalls angedeutet (ebenda, S. 88). In Tab. 4 führe ich die formale Argumentationsanalyse am Beispiel der Ad-Hoc Empfehlung zur Verteilung von Impfstoffen durch.

**Table 4:** 

Ground	„Anfängliche Knappheit von COVID-19-Impfstoffen erfordert Auswahlentscheidungen darüber, wer zuerst geimpft werden soll. Priorisierungsentscheidungen berühren ethisch wie rechtlich elementare Fragen, insbesondere des Gesundheits- und Lebensschutzes jedes Einzelnen sowie der Gerechtigkeit und der Solidarität zwischen allen betroffenen Mitgliedern einer Gesellschaft“ (Deutscher Ethikrat 2020b, S. 2)
Claim	„Leitend für die künftige detaillierte Empfehlung einer Priorisierung sind die (…) ausgeführten ethischen und rechtlichen Prinzipien sowie folgende konkrete Impfziele: • Verhinderung schwerer COVID-19-Verläufe (Hospitalisation) und Todesfälle • Schutz von Personen mit besonders hohem arbeitsbedingten SARS-CoV-2-Expositionsrisiko (berufliche Indikation) • Verhinderung von Transmission sowie Schutz in Umgebungen mit hohem Anteil vulnerabler Personen und in solchen mit hohem Ausbruchspotenzial • Aufrechterhaltung staatlicher Funktionen und des öffentlichen Lebens“ „Infolgedessen sind diejenigen prioritär zu impfen, die bei einer Erkrankung an COVID-19 das höchste Risiko für Tod und schwere Erkrankung tragen“ (Deutscher Ethikrat 2020b, S. 3)
Warrant	„Den Ausgangspunkt bildet die Selbstbestimmung (‚Autonomie‘) jedes Einzelnen. Impfungen setzen prinzipiell eine aufgeklärte, freiwillige Zustimmung voraus“ (Deutscher Ethikrat 2020b, S. 2). „Zugleich ist der ethische Grundsatz der Nichtschädigung bzw. des Integritätsschutzes berührt. Alle Priorisierungsentscheidungen müssen sich daran messen lassen, ob sie schwere Schädigungen verhindern helfen – Schäden, denen mittels Selbstschutz der zu impfenden Personen durch Immunität vorgebeugt werden kann, aber auch Schäden, die aus einem mangelnden Fremdschutz für andere resultieren und deshalb durch eine Unterbindung der Transmission von Krankheitserregern abgewendet werden können“ (ebenda, S. 2). „Demgegenüber muss der ethische Grundsatz der Wohltätigkeit, insbesondere im Sinne der individuellen ärztlichen Fürsorgepflicht bei Priorisierungsentscheidungen im Konfliktfalle zurücktreten. Üblicherweise sieht sich die Medizin in der Pflicht, das Wohl ihrer Patient*innen bestmöglich zu fördern. Bei starker Knappheit von geeigneten Mitteln ist das kaum möglich. Hier geht es um die ausreichende Basisversorgung möglichst vieler und nicht um die maximale Bestversorgung einiger weniger“ (ebenda, S. 2). „Von zentraler Bedeutung für Priorisierungsentscheidungen sind der ethische Grundsatz der Gerechtigkeit und die grundlegende Rechtsgleichheit. Sie verbieten nicht nur bestimmte inakzeptable Differenzierungskriterien, sondern verlangen im Grundsatz, (wesentlich) Gleiche gleich und (wesentlich) Ungleiche ungleich zu behandeln. Gleiche Gefährdungslage begründet deshalb gleichen Versorgungsanspruch. Umgekehrt gilt: Ungleiche Gefährdungslage rechtfertigt und erfordert ungleiche Versorgung“ (ebenda, S. 2). „Dieser Gerechtigkeitsaspekt ist eng verknüpft mit dem ethischen Grundsatz der Solidarität: Solidarbereite Personen zeigen Verantwortung gegenüber stärker gefährdeten Personen und stellen dafür den eigenen Anspruch auf ihren raschen Gesundheitsschutz – zumindest zeitweilig – zurück“ (ebenda, S. 2)
Backing	Keine Berücksichtigung
Rebuttal	Keine Berücksichtigung
Qualifier	„Ethisch und rechtlich zulässige Priorisierungsentscheidungen müssen zudem formalen und prozeduralen Mindestanforderungen genügen. Sie müssen auf der aktuellen und kontinuierlich aktualisierten medizinisch-naturwissenschaftlichen Faktenlage basieren; sie müssen sowohl verfassungskonform wie unter Anwendung der skizzierten ethischen Grundsätze überzeugend begründet sein, und sie müssen unter Einbeziehung aller relevanten Betroffenen weitest möglich konsentiert, in transparenten Verfahren öffentlich kommuniziert und gesetzlich abgesichert sein“ (Deutscher Ethikrat 2020b, S. 3)

Die Ad-Hoc Empfehlung zur Verteilung von Impfstoffen erschien im Rahmen eines gemeinsamen Positionspapiers, erarbeitet in Zusammenarbeit mit der Ständigen Impfkommission des Robert Koch-Instituts und die Nationale Akademie der Wissenschaften Leopoldina. Ziel der Verfasser ist es, zu klären wie der Zugang zu COVID-19-Impfstoffen auf ethisch, rechtlich und praktisch sinnvolle Weise geregelt werden kann. Wie für Ad-Hoc Empfehlungen typisch fehlt ein normativer Reflexionsteil. Mithin drückt die normative Forderung diejenigen prioritär zu impfen, die bei einer Erkrankung an COVID-19 das höchste Risiko für Tod und schwere Erkrankung tragen, eine Konsensposition der beteiligten Organisationen aus. Der Verzicht auf einen normativen Reflexionsteil schlägt sich auch auf die formale Argumentationsstruktur nieder. Die formalen Kriterien für robuste Argumente nach Toulmin werden nicht erfüllt. Durch den Verzicht ethischen Abwägens werden weder mögliche Gegenargumente (rebuttals) berücksichtigt, noch werden Gründe genannt, welche die normativen Schlussregeln begründen (backing). Als Informationsgrundlage für Gewissensentscheidungen der politischen Entscheidungsträger ist dieses Dokument daher nur bedingt geeignet. Wenn vom Ethikrat eine legitimierende Funktion für politische Willensbildungsprozesse im Sinne der Diskurstheorie von Habermas (1991, 1992) ausgehen soll, dann ist die Legitimation durch die Autorität der beteiligen Organisationen und ihrer Mitglieder (Deutscher Ethikrat, Leopoldina, Ständigen Impfkommission des Robert Koch-Instituts) für sich genommen keine hinreichende Grundlage ethischer Politikberatung. Autorität beansprucht in der Diskurstheorie nur das überzeugendere Argument, unabhängig von wem es formuliert wird. Arbeiten ethische Gremien jedoch im Sinn einer „Black Box“, dann entspricht dies nicht dem Modell einer aufgeklärten Gesellschaft, sondern eher dem Modell obrigkeitsstaatlichen Denkens. Zur Verteidigung des Deutschen Ethikrats lässt sich jedoch anführen, dass es am 18. November 2020 eine Online-Veranstaltung mit dem Titel „Wer zuerst? Verteilung von Impfstoffen gegen SARS-CoV-2“ gab. Die Veranstaltung bestand aus drei Vorträgen und einer Podiumsdiskussion. Der Vortrag von Alena Buyx, Vorsitzende des Deutschen Ethikrates, gibt weitestgehend den Stand der Ad-Hoc Empfehlung wieder. Im Vortrag von Mariângela Simão, stellvertretende WHO-Generaldirektorin für Arzneimittel und Gesundheitsprodukte, geht es primär um die Verteilung der Impfstoffe aus einer globalen Perspektive. Der Vortrag von Christiane Woopen, Vorsitzende der European Group on Ethics in Science and New Technologies, diskutiert in ihrem Vortrag fünf Ansätze zur Impfstoffverteilung: (1) Lotterie, (2) Wer zuerst kommt, der mahlt zuerst, (3) Soziale Rolle/Funktion in der Gesellschaft, (4) Utilitarismus, (5) Bedürftigkeitsorientierter Ansatz. Durch die Kürze des Vortrags werden zentrale Aspekte apodiktisch abgehandelt. Eine richtige Diskussion, in der die einzelnen normativen (Gegen‑)Position kritisch gewürdigt und diskutiert werden, findet nur im Ansatz statt. Hier ein Beispiel aus dem Vortrag von Woopen:

Wir könnten auch danach streben, die Vorteile, den Nutzen zu maximieren. Bei einem utiliaristischen Ansatz nutzt man als Grundlage vor allem die Anzahl von geretteten Leben, es sollen also so viele Menschen wie möglich geschützt werden. Man kann auch konkreter vorgehen und sagen: so viele Lebensjahre wie möglich. Damit hätten die Jüngeren eine größere Chance, als Erste dranzukommen. Es wird oft kritisiert, dass ein solcher utilitaristischer Ansatz die Menschenwürde verletzt. Jeder Mensch hat den gleichen Wert, und dreißig Leben oder Lebensjahre zu retten ist nicht ethisch besser als drei. Wenn in einer Pandemie diejenigen mit prioritärem Status, die also ein besonders hohes Risiko haben, dass sie schwer erkranken oder sterben, dann heißt das nicht, dass sie wertvoller sind als andere. Es wird nur die Tatsache anerkannt, dass andere warten können. (Woopen 2020, S. 8)

Nach Woopen ist eine utilitaristische Verteilung des Impfstoffs unmoralisch, weil dreißig Lebensjahre zu retten nicht besser sei als das Leben eines alten Menschen um drei Jahre zu verlängern. Jeder Mensch ist gleich viel wert. Aus einer gerechtigkeitsethischen Perspektive kann dies durchaus hinterfragt werden. Diejenigen, die durch eine vorzeitige Impfung am meisten Lebensjahre gewinnen, haben tendenziell bislang am wenigsten lange gelebt. Verdienen es die jungen Menschen nicht, dass ihr Leben zunächst gerettet wird, weil sie bisher am wenigsten lange gelebt haben? Ist eine Lebensverlängerung hochbetagter Personen, die nur noch im Bett dahinsiechen, wirklich äquivalent zum Dasein junger Menschen, die ihr Leben mit all ihren Wünschen und Träumen noch vor sich haben?

Die Frage nach der Impfreihenfolge ist aus einer ethischen Perspektive mindestens so komplex wie die normativen Konflikte im Zusammenhang mit der Ausstellung von Immunitätsbescheinigungen. Die Ad-Hoc Empfehlung und ein hierzu abgehaltenes Online-Seminar (vgl. Woopen 2020) werden den komplexen normativen Fragen im Zusammenhang mit der Impfreihenfolge nicht gerecht, weil das Thema weder in der Tiefe noch in der Breite umfassen behandelt wird.

Im Beitrag „Vaccine distribution ethics: monotheism or polytheism?“ plädieren die Autoren Alberto Giubilini, Julian Savulescu und Dominic Wilkinson (2020) für eine offene ethische Debatte, die mehr Faktoren berücksichtigt als nur das Bedürftigkeitsprinzip, wie es der Deutsche Ethikrat nahelegt. Selbst wenn man vom Prinzip der Bedürftigkeit als zentralem Kriterium zur Impfstoffverteilung ausgeht, greifen die vorgelegten Analysen zu kurz, da die Anzahl der Todesfälle von vielen Faktoren bestimmt wird, die eine Betrachtung jenseits des bloßen Infektionsrisikos erfordert. Wie wahrscheinlich ist es zum Beispiel, dass verschiedene Gruppen das Virus verbreiten? Wie wirksam ist ein Impfstoff bei verschiedenen Gruppen? Wie wirksam ist ein Impfstoff bei der Verhinderung einer Ansteckung im Vergleich zur Verhinderung, dass Einzelpersonen erkranken?

Die normative Frage bei der Impfstoffverteilung lautet nicht, ob wir diskriminieren sollten, sondern ob die Grundlage der Diskriminierung ethisch vertretbar ist. Da es sich hier um Gewissensentscheidungen handelt, für die es oft keine eindeutige normative Basis gibt, wäre es aus einer demokratietheoretischen Perspektive wünschenswert, wenn die Pluralität an Weltanschauungen in den Erörterungen des Deutschen Ethikrats umfassend berücksichtigt würde.

## Resümee

Der Deutsche Ethikrat hat die Ressourcen, um im Rahmen von wissenschaftlichen, rechtlichen und ethischen Expertisen Impulse für den politischen Willensbildungsprozess zu geben. Dies setzt jedoch voraus, dass dieses Gremium jedem Verdacht erhaben ist, parteiisch zugunsten bestimmter Position Stellung zu beziehen. Gerade für normative Stellungnahmen ist der Grat zwischen ethischer Beratung und politischem Aktivismus ein enger. Weder politischer Aktivismus im Sinne einer einseitigen politischen Beeinflussung politischer Entscheidungsträger, noch die Instrumentalisierung der Ethik als Propagandainstrument sind mit dem Mandat des Deutschen Ethikrats vereinbar. Gegen den Vorwurf unzulässiger Einflussnahme kann sich der Ethikrat vor allem durch zwei Maßnahmen absichern. Zum einen indem er die Pluralität normativer Perspektiven in der Gesellschaft intern abbildet, zum anderen, indem er normative Positionen intersubjektiv nachprüfbar und möglichst umfassend begründet. Letzteres war Gegenstand der Untersuchung in diesem Beitrag.

Der Argumentationsanalyse nach Toulmin hat gezeigt, dass hinsichtlich der formalen Struktur der Stellungnahmen und Empfehlungen Verbesserungspotential besteht. Im Rahmen der Stellungnahmen zu Immunitätsbescheinigungen hätten die Positionen noch stärker auf wissenschaftliche Studien und gesetzliche Normen verweisen können, um so den Argumenten ihren zum Teil spekulativen Charakter zu nehmen. Die normative Position, die sich gegen Immunitätsbescheinigungen ausspricht, geht darüber hinaus nur unzureichend auf die Argumente der Befürworter von Immunitätsbescheinigungen ein, um sie zu Gunsten ihrer eigenen Position zu entkräften. Obwohl im Rahmen der Stellungnahme die formalen Kriterien für robuste Argumente erfüllt werden, ist der Begründungszusammenhang nicht immer in der Tiefe gegeben, sodass kritische Rückfragen auf der veröffentlichten Textgrundlage nicht beantwortet werden können.

Im Rahmen der Ad-Hoc Empfehlung zur Impfreihenfolge wird, wie für dieses Publikationsformat typisch, auf einen Reflexionsteil verzichtet. Der Verzicht mag den Autoren gerechtfertigt erscheinen, da in diesem Format Konsenspositionen wiedergegeben werden. Durch den Verzicht ethischer Reflexion werden die formalen Kriterien für robuste Argumentation nach Toulmin (2003) jedoch verletzt. In der Konsequenz handelt es sich bei der Ad-Hoc Empfehlung auch weniger um eine Argumentation, sondern eher um die Verlautbarung einer Mehrheitsmeinung der beteiligten Institutionen und Autoren. Durch den Verzicht einer ethischen Erörterung fällt es schwer zu ermessen, inwieweit dieses Dokument noch geeignet ist, den Rezipienten (im Sinne einer Beratung) in die Lage zu versetzen, *sich selbst* ein eigenes normatives Urteil zu den betreffenden Wertkonflikten zu bilden. Außerdem gilt es zu bedenken, dass es den beteiligten Autoren durch das Konsensvotum vermutlich nicht gelingt, die Vielfalt normativer Positionen in der Gesellschaft zu der behandelten kontroversen Thematik zu repräsentieren. Der Beitrag von Giubilini et al. (2020) zeigt, dass die Schlussregel, die Impfreihenfolge nach dem Prinzip der Bedürftigkeit zu regeln, nicht unumstritten ist, wie es die Ad-Hoc Empfehlung suggeriert wird. In der Tat ist die Frage nach der Impfreihenfolge normativ sehr komplex und das bestehende naturwissenschaftlich-technische Wissen zur Covid-19 Pandemie lückenhaft. Statt einer Ad-Hoc Empfehlung, die wie eine „black box“ erscheint, wäre es vielleicht angemessener gewesen, eine Art Reflexionshilfe für politische Entscheidungsträger zu veröffentlichen. Die Reflexionshilfe hätte zunächst einen Überblick über den rechtlichen und technisch-naturwissenschaftlichen Stand geben und anschließend eine Art Checkliste mit Punkten bereitstellen können, die für die weitere ethische Reflexion und Urteilsbildung berücksichtigt werden sollten.

Bieber (2013) warnt, dass ethische Beratungsorgane zur Legitimation von politisch vorgefassten Entscheidungen instrumentalisiert werden können. Der vorliegende Beitrag hat einige Schwachstellen in der formalen argumentativen Qualität in den Veröffentlichungen des Deutschen Ethikrats zur Covid-19 Pandemie offengelegt. Aus meiner Sicht ist es geboten, die Unabhängigkeit und Pluralität des Deutschen Ethikrat sicherzustellen und mehr Ressourcen für die Qualitätssicherung von Stellungnahmen und Ad-Hoc Empfehlungen aufzuwenden. Die diskursive Qualität ist die zentrale Legitimationsquelle von politik- und gesellschaftsberatende Ethikkommissionen. Werden Zweifel an der Redlichkeit ethisch beratender Institutionen laut, wie etwa im Falle des Abschlussberichts der Ethikkommission Sichere Energieversorgung^1^, schadet dies nicht nur dem Ansehen ethischer Beratungsorgane, sondern wirkt sich auch negativ auf das Vertrauen in die Demokratie und langfristig auf die Stabilität des politischen Systems aus.

## Footnotes

1 Nach der Katastrophe in Fukushima im Mai 2011 wurde vom Bundeskanzleramt eine Ethikkommission Sichere Energieversorgung eingesetzt. Nach nur sechswöchiger Arbeitszeit veröffentlichte die Kommission ihren Abschlussbericht, in der sie einstimmig für ein Ende der nuklearen Energieerzeugung in Deutschland votiert. Angesichts der gegenwärtigen Pandemie- und Klimapolitik und der wachsenden Zweifel an der Unabhängigkeit der Wissenschaft veröffentlichte Thess (2021), der an der Universität Stuttgart eine Professur für Energiespeicherung vertritt, zum 10. Jahrestag des Abschlussberichts einen offenen Brief. In diesem Brief kritisiert er, dass die Verfasser des Abschlussberichts die Leitlinien guter wissenschaftlicher Praxis, sowie die Leitlinien zur Politikberatung der Berlin-Brandenburgischen Akademie der Wissenschaften verletzt hätten.
